# CYP2W1 Is Highly Expressed in Adrenal Glands and Is Positively Associated with the Response to Mitotane in Adrenocortical Carcinoma

**DOI:** 10.1371/journal.pone.0105855

**Published:** 2014-08-21

**Authors:** Cristina L. Ronchi, Silviu Sbiera, Marco Volante, Sonja Steinhauer, Vanessa Scott-Wild, Barbara Altieri, Matthias Kroiss, Margarita Bala, Mauro Papotti, Timo Deutschbein, Massimo Terzolo, Martin Fassnacht, Bruno Allolio

**Affiliations:** 1 Endocrine and Diabetes Unit, Department of Internal Medicine I, University Hospital, University of Wuerzburg, Wuerzburg, Germany; 2 Department of Oncology, University of Turin, San Luigi Hospital, Turin, Italy; 3 Institute of Pathology, University of Wuerzburg, Wuerzburg, Germany; 4 Comprehensive Cancer Center Mainfranken, University Hospital, University of Wuerzburg, Wuerzburg, Germany; 5 Division of Internal Medicine I, University of Turin, San Luigi Hospital, Turin, Italy; Istituto dei tumori Fondazione Pascale, Italy

## Abstract

**Background:**

Adrenocortical tumors comprise frequent adenomas (ACA) and rare carcinomas (ACC). Human cytochrome P450 2W1 (CYP2W1) is highly expressed in some cancers holding the potential to activate certain drugs into tumor cytotoxins.

**Objective:**

To investigate the CYP2W1 expression in adrenal samples and its relationship with clinical outcome in ACC.

**Material and Methods:**

CYP2W1 expression was investigated by qRT-PCR in 13 normal adrenal glands, 32 ACA, 25 ACC, and 9 different non-adrenal normal tissue samples and by immunohistochemistry in 352 specimens (23 normal adrenal glands, 33 ACA, 239 ACC, 67 non-adrenal normal or neoplastic samples).

**Results:**

*CYP2W1* mRNA expression was absent/low in normal non-adrenal tissues, but high in normal and neoplastic adrenal glands (all *P<0.01* vs non-adrenal normal tissues). Accordingly, CYP2W1 immunoreactivity was absent/low (H-score 0–1) in 72% of non-adrenal normal tissues, but high (H-score 2–3) in 44% of non-adrenal cancers, in 65% of normal adrenal glands, in 62% of ACAs and in 50% of ACCs (all *P<0.001* vs non-adrenal normal tissues), being significantly increased in steroid-secreting compared to non-secreting tumors. In ACC patients treated with mitotane only, high CYP2W1 immunoreactivity adjusted for ENSAT stage was associated with longer overall survival and time to progression (*P<0.05* and *P<0.01,* respectively), and with a better response to therapy both as palliative (response/stable disease in 42% vs 6%, *P<0.01*) or adjuvant option (absence of disease recurrence in 69% *vs* 45%, *P<0.01*).

**Conclusion:**

CYP2W1 is highly expressed in both normal and neoplastic adrenal glands making it a promising tool for targeted therapy in ACC. Furthermore, CYP2W1 may represent a new predictive marker for the response to mitotane treatment.

## Introduction

Most tumors of the adrenal cortex are adenomas (ACA), whereas malignant carcinomas (ACC) are rare. Both types can be either endocrinologically silent or hormonally active, with steroid hormone production being present in at least 60% of ACCs [1]. Malignant tumors are highly aggressive with poor prognosis and an overall survival rate at 5 years of only 25–50% in most series [2], [3], [4]. Thus, malignant adrenocortical tumors remain a major therapeutic challenge and new targets for treatment are urgently needed [5].

Cytochrome P450 enzymes are a large family of hemeproteins with several members being involved in specific steps of steroidogenesis (for instance CYP2B1) or in the metabolism of drugs and other xenobiotics (e.g. CYP2D6, CYP3A4, CYP2C9). The human cytochrome P450 2W1 (CYP2W1) enzyme has been originally classified as “orphan” because information regarding its physiologic enzymatic function was lacking. CYP2W1 shares some substrate affinity and sequence homology with other P450 enzymes, such as CYP1A1, which is involved in the metabolism of polycyclic aromatic hydrocarbons and associated with higher lung cancer risk [6]. Recent studies using recombinant CYP2W1 demonstrated broad catalytic activity towards several chemicals, including bioactivation of procarcinogens like aflatoxin B1, polycyclic aromatic hydrocarbon dihydrodiols and aromatic amines [7], [8], [9]. Furthermore, it seems to be involved in the oxydation of lysophospholipids and fatty acids [10].

CYP2W1 mRNA and/or protein are known to be highly expressed during foetal life and in some cancers, particularly colon cancer, but also adrenocortical, lung and gastric cancers [11], [12], [13], [14], [15]. Only limited data is available on CYP2W1 mRNA expression in various normal adult human tissues [16] and protein expression has not been specifically investigated in these tissues.

Taken together, these features make CYP2W1 a potential tool for cancer therapy [6], [15], as it may activate specific prodrugs into toxic compounds selectively in cancer cells.

No detailed information on CYP2W1 expression in normal and neoplastic adrenal glands has been reported. We therefore analysed CYP2W1 expression in a large series of adrenal tissues with the major aim to assess its prognostic role in adrenocortical malignancy and to evaluate its predictive potential for the response to different treatments.

## Materials and Methods

### Tissue samples

Seventy fresh frozen adrenal tissues (21 normal adrenal glands, 32 ACA, and 25 ACC) were investigated for evaluation of CYP2W1 mRNA levels. The adrenocortical tumors included both hormonally active (n = 34) and endocrinologically silent masses (n = 23). A total of 16 other normal non-adrenal tissues, consisting of colon (n = 4), liver (n = 4), kidney (n = 3), thyroid (n = 2), lung (n = 2), and stomach (n = 1) were used as controls.

A total of 429 paraffin-embedded specimens, including 124 standard full slides and 305 samples assembled into six tissue microarrays (TMAs) from two centers were available for immunohistochemistry. TMA samples were included in the analysis only if two or more evaluable cores per patient were available after the staining procedure. Thus, the final series included a total of 370 tissue samples as follows: 18 normal adrenal glands and 5 adrenal hyperplasia, 39 ACA, 240 ACC, 34 non-adrenal normal tissues, such as uterus (n = 4), stomach (n = 4), ovary (n = 3), tonsil (n = 3), kidney (n = 3), liver (n = 2), colon (n = 2), pancreas (n = 2), prostate (n = 2), lung (n = 2), thyroid (n = 2), breast (n = 2), spleen (n = 1), tongue (n = 1), ileum (n = 1), and 34 non-adrenocortical tumors, such as pheochromocytoma (n = 9), colon cancer (n = 4), pancreas cancer (n = 3), breast cancer (n = 3), hepatocellular carcinoma (n = 3), ovary cancer (n = 2), prostate cancer (n = 2), pulmonary adenocarcinoma (n = 2), renal cell carcinoma (n = 2), testicular carcinoma (n = 1), lung cancer (n = 1), melanoma (n = 1), and lymphoma (n = 1). Among the ACC samples, 196 were obtained from surgery of the primary tumor, 26 from local recurrences and 18 from distant metastases.

A total of 25 adrenocortical tumor samples with both frozen tissue and paraffin slides (8 adenomas and 17 carcinomas) were used for the comparison between CYP2W1 mRNA levels and immunoreactivity.

### Patients and clinical annotations

Clinical parameters, such as sex, age at diagnosis, date of surgery, tumor size, and results of hormone analysis, and in case of ACC, tumor stage according to the European Network for the Study of Adrenal Tumors (ENSAT) classification [17], Weiss score, Ki67 index, presence and number of distant metastasis, and detailed follow-up information were collected through the German ACC and the ENSAT Registry (www.ensat.org/registry, [18]). Malignancy and hormonal hypersecretion were defined according to established clinical, biochemical, and morphological criteria [19]. For the correlation with clinical data, we considered only ACC samples obtained from primary surgery (n = 196). Concerning the different treatment modalities, 39 patients did not receive any therapy after surgery (follow up only), 72 patients were treated with mitotane only (in an adjuvant, n = 36, or in a palliative setting, n = 36), 64 patients underwent different systemic cytotoxic chemotherapy regimens (51 of whom in combination with mitotane), while for 21 patients such data were not available. Concerning mitotane treatment, the median duration of mitotane therapy was of 6 months (range 1–71 months) in patients treated with palliative intent and 41 months (range 5–161 months) in those treated adiuvantly. The dosage of mitotane ranged between 1 g and 6 g per day aiming at target serum concentrations between 14 and 20 mg/l. Among the 61 patients treated with systemic chemotherapy, 40 received a platinum-based treatment in first line (in 30 as part of the etoposide+doxorubicin+cisplatin (EDP) regimen), 14 streptozotocin [20]. The objective response to therapy was retrospectively evaluated in a total of 124 ACC patients.

The study was approved by the ethics committee of the University of Würzburg (No. 93/02 and 88/11) and written informed consent was obtained from all patients.

### Gene expression analysis

*CYP2W1* mRNA expression levels were investigated by real-time quantitative PCR (qRT-PCR). In brief, RNA was isolated from fresh frozen tissue samples using the RNeasy Lipid Tissue Minikit (Qiagen, Alameda, CA, USA) and reverse transcribed using the QuantiTect Reverse Transcription Kit (Qiagen). Two predesigned Taqman Gene Expression assays for *CYP2W1* (Hs00908623_m1, Exon boundary 7–9, and Hs00214994_m1, Exon boundary 2–3) were purchased from Applied Biosystems (Darmstadt, Germany). Endogenously expressed *β-actin* (Hs9999903_m1) was used for normalization. 40 ng cDNA was used for each PCR reaction and each sample was performed in duplicate. Transcript levels were determined using the TaqMan Gene Expression Master Mix (Applied Biosystems), the CFX96 real-time thermocycler (Bio-rad, Hercules, CA, USA) and Bio-Rad CFX Manager 2.0 software. Cycling conditions were 95°C for three min followed by 50 cycles of 95°C for 30 sec, 60°C for 30 sec, and 72°C for 30 sec. Using the ΔCT method [21], the gene expression levels were normalized to those of *β-actin*, as previously described [22].

### Immunohistochemistry

For our analysis, we selected a highly specific rabbit antibody (Ab) raised against the N-Terminal region of CYP2W1, which is well suitable for immunohistochemistry (**Ab #1**, PA5-14900, Thermo Fisher Scientific Inc., Waltham, MA USA, epitope: AQDPSPAARWPP). In particular, **Ab #1** showed at the specificity analysis the maximal specificity among the components of the CYP family (100% specificity for the CYP2W1 isoform 1, Q8TAV3, UNIPROTKB, www.uniprot.org, and CLC Sequence Viewer, Version 7, www.clcbio.com/products/clc-sequence-viewer). Moreover, we tested different available polyclonal antibodies against CYP2W1 with Western Blot analysis using microsomal fractions of HEK293 cell lines transfected with pCMV4-CYP2W1 vector (kindly provided by Prof. Ingelman-Sundberg, Karolinska Institute, Sweden [15]) as positive control. In particular, we compared **Ab #1** (diluition 1:50) with a rabbit Ab raised against the C-Terminal region used in several previous publications and kindly provided by Prof. Ingelman-Sundberg (**Ab #2**, diluition 1∶1000, epitope: TMRPRAQALCAVPRP) [11], [14], [15], [23] and with another rabbit Ab raised against an internal peptide (Santa Cruz Biotechnology Inc., Dallas, Texas, USA, H-175, sc-98920, 1∶200, 175 aminoacids, [24]). With all three antibodies we detected a specific band of 54 KDa corresponding to CYP2W1 (and at least one additional band at 70 KDa, ***Figure S1***).

#### Western Blot analysis

proteins were extracted with RIPA buffer. Electrophoresis of equal amounts of lysates was performed in a bis-tris gel system and protein transferred onto a PVDF membrane by a tank-blotting procedure. The membrane was blocked with 5% skim milk powder followed by overnight probing with the different antibodies at 4°C (see above). After three washes, the membrane was incubated with horseradish peroxidase-labeled goat anti-rabbit IgG as secondary antibody (1∶5000). The antigen-antibody complex was visualized by enhanced chemiluminescence using an Amerscham ECL reagent. Normalization of protein levels was performed by re-probing the blot with an antibody recognizing human β-actin (Sigma-Aldrich, St. Louis, USA, 1∶5000). Autoradiographs were scanned, and the quantification of individual bands was measured using Image J software (NCBI).

#### CYP2W1 immunostaining

TMA and full sections were deparaffinized and immunohistochemical detection was performed using an indirect immunoperoxidase technique after high temperature antigen retrieval in 10 mM citric acid monohydrate buffer (pH 6.5) in a pressure cooker for 13 min. Blocking of unspecific protein-antibody interactions was performed with 20% human AB serum in PBS for 1 h at RT. Primary antibody was the **Ab #1**, used at a dilution of 1∶50 at 4°C overnight together with the N-Universal Negative Control Anti-Rabbit (Dako, Glostrup, Denmark). For comparison, **Ab #2** was also used in a subset of 25 samples (including 8 different normal tissues and 17 different tumors) at a dilution of 1∶500. Signal amplification was achieved by En-Vision System Labeled Polymer-HRP Anti-Rabbit (Dako) for 40 min and developed for 10 min with DAB Substrate Kit (Vector Laboratories, Burlingame, CA, USA) according to the manufacturer’s instructions. Nuclei were counterstained with Mayer’s hemalaun for 2 min. For positive controls, sections with colon cancer, pancreas cancer and prostate cancer were chosen, while cells of the tumor stroma served as internal negative control.

#### Microscopic analysis of CYP2W1 immunoreactivity

**Figure 1:**
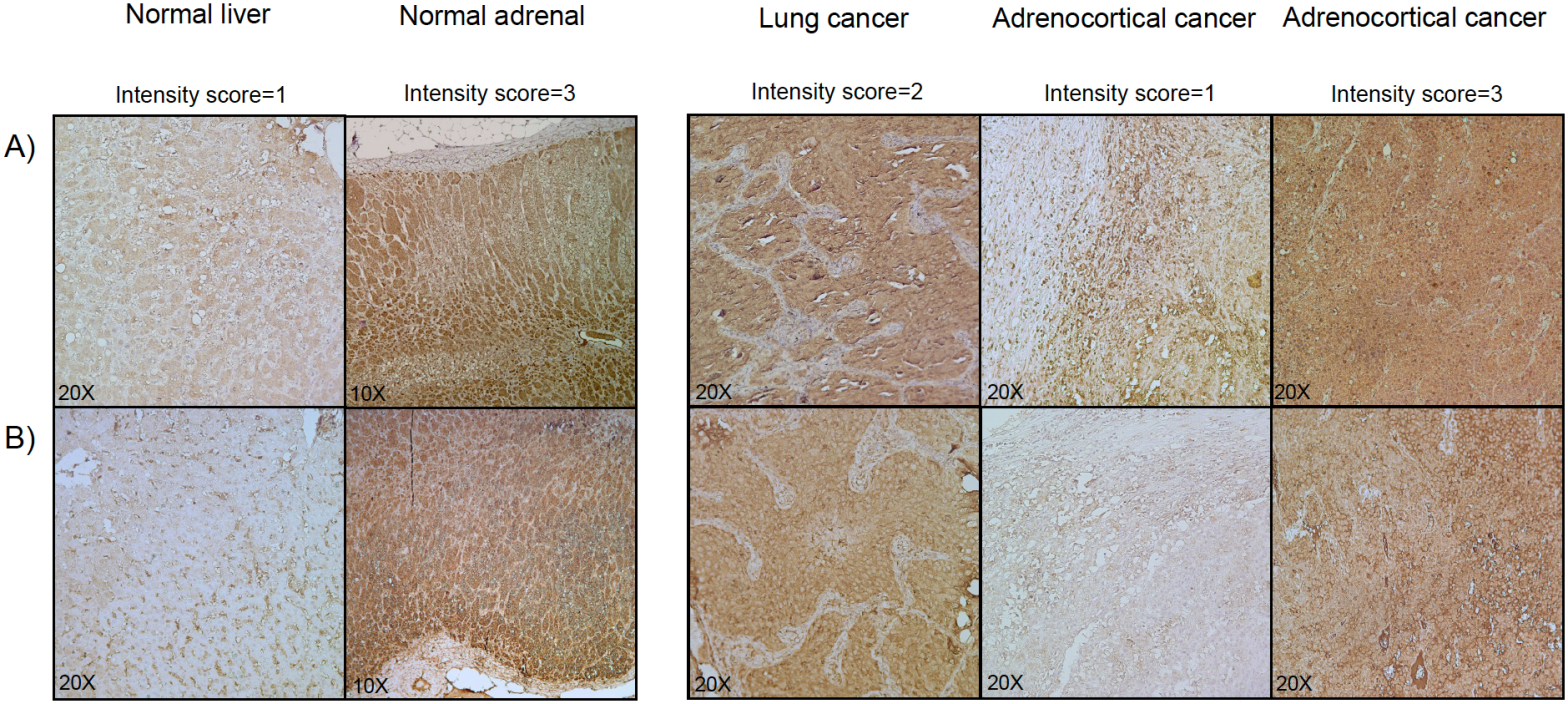
CYP2W1 immunohistochemistry. Examples of CYP2W1 immunostaining in normal tissue (i.e. liver and adrenal gland) and neoplastic tissues (i.e. lung cancer and adrenocortical carcinoma). A: Staining with a polyclonal antibody from Thermo Scientific (**Ab #1**); B: Staining with a polyclonal antibody provided by the Karolinska Institute [11], [14], [23] (**Ab #2**). Magnification 20X and 10X.

All slides were analyzed independently by two investigators blinded to clinical information (C.L.R. and So. St.). Both nuclear and cytoplasmic staining was evaluated, and staining intensity was graded as negative (0), low (1), medium (2), or strong (3). The percentage of positive tumor cells was calculated for each specimen and scored 0 if 0% were positive, 0.1 if 1–9% were positive, 0.5 if 10–49% were positive and 1 if 50% or more were positive. A semiquantitative H-score was then calculated by multiplying the staining intensity grading score with the proportion score as described previously [25]. Where discrepancies were observed, results were jointly assessed by both investigators and the final score was formed by consensus. Inter-observer agreement was strong with a Fleiss k-coefficient of 0.878 and a Pearson’s correlation coefficient of 0.70 (95% CI 0.58–0.78). Some examples of the CYP2W1 staining in normal and neoplastic tissues are given in ***Figure 1***.

### Statistical analysis

The Fisher’s exact test or the Chi-square test was used to investigate dichotomic variables, while a two-sided *t* test (or non-parametric test) was used to test continuous variables. A non parametric Kruskal-Wallis test, followed by Bonferroni *post-hoc* test, was used for comparison among several groups for non-normal distributed variables. Correlations and 95% confidence intervals (95% CI) between different parameters were evaluated by linear regression analysis. Overall survival (OS) was defined as the time from the date of primary surgery to specific death or last follow-up. Disease-free survival (DFS) was defined as the time from the date of complete tumor resection to the first radiological evidence of disease relapse or death. Time to progression was defined as the time from begin of treatment until first radiological evidence of any kind of disease progression. All survival curves were obtained by Kaplan-Meier estimates and the differences between survival curves were assessed by the log-rank (Mantel-Cox) test. In this context, the RNA expression was considered as a categorical value (cut-off value for this data set: median value +2SD). A multivariate regression analysis was performed by Cox proportional hazard regression model to identify those factors that might independently influence survival. Statistical analyses were made using GraphPad Prism (version 5.0, La Jolla, CA, USA) and SPSS Software (PASW Version 21.0, SPSS Inc., Chicago, IL, USA). P values *<0.05* were considered as statistically significant.

## Results

### CYP2W1 is highly expressed in normal and neoplastic adrenal glands

**Figure 2:**
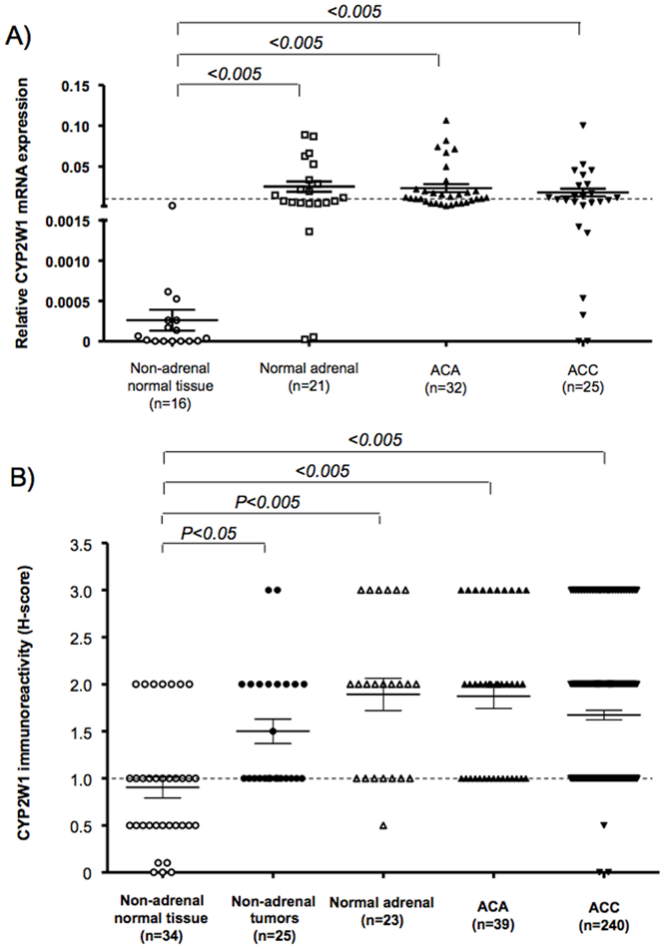
CYP2W1 expression in adrenal and non-adrenal tissues. A: Relative mRNA expression levels (normalized to *β-actin* using the ΔCT method, [21]). B: CYP2W1 immunoreactivity evaluated as H-score (for details see Material and methods section). *P* values were evaluated by non parametric Kruskal-Wallis test and Bonferroni *post-hoc* test. ACA = adrenocortical adenoma, ACC = adrenocortical carcinoma.

*CYP2W1* mRNA expression was generally very low in non-adrenal normal tissues (mean ΔCT±SD: 0.00012±0.000059), but well detectable in normal adrenal glands (0.035±0.032, *P<0.005,* ***Figure 2A***). These results were consistent for second *CYP2W1* assay for qRT-PCR (data not shown). Adrenocortical tumors also showed a strong mean *CYP2W1* expression (ACA = 0.024±0.027 and ACC = 0.018±0.023, ***Figure 2A***). However, a subgroup of ACCs showed low *CYP2W1* levels in comparison with NA and ACA. No statistically significant correlation was observed between mRNA levels and histological or clinical parameters.

In immunostaining, CYP2W1 expression was mainly localised in the cytoplasm and was homogeneously distributed, with a median percentage of positive cells of 80% (range 40 to 100%). For samples studied both as full slides and in TMA the results obtained in standard full slides were consistent with those obtained in the TMA. In concordance with the mRNA analysis, in the majority of the non-adrenal normal tissues, CYP2W1 immunoreactivity was absent or low (H-score 0–1 in 72%, 2 in 25% and 3 in 3% of cases), whereas it was significantly higher in our series of non-adrenal cancers (H-score 0–1 in 56%, 2 in 36% and 3 in 8% of cases, *P<0.05 vs* normal tissues, ***Figure 2B***). Surprisingly, CYP2W1 immunoreactivity was even higher in normal adrenal glands (H-score 0–1 in 35%, 2 in 43% and 3 in 22% of cases, *P<0.005 vs* non-adrenal normal tissues), being slightly more intense in the zona reticularis and fasciculata than in the zona glomerulosa (***Figure 1***). Of note, also the adrenal medulla stained positive for CYP2W1. The CYP2W1 immunoreactivity in both benign and malignant adrenocortical tumors was similar to that in normal adrenal glands (***Figure 2B***). A good correspondence between CYP2W1 mRNA expression levels and immunoreactivity was observed in the 25 adrenocortical tumor samples evaluated with both methods (*P<0.001* by Chi-Square test per trend, ***Figure S2***).

Very similar results in terms of staining intensity were obtained in the 25 samples (including both normal and neoplastic tissues) evaluated with both antibodies (**Ab #1** and **Ab #2**). An example of the staining in normal liver and normal adrenal gland with the two antibodies is shown in ***Figure 1***.

Among the ACCs, no difference in CYP2W1 expression was observed between primary tumors (n = H-score 0–1 in 49%, 2 in 33% and 3 in 18% of cases), local recurrences (H-score 0–1 in 41%, 2 in 32% and 3 in 27% of cases), and metastatic tissues (H-score 0–1 in 57%, 2 in 31% and 3 in 12% of cases).

### CYP2W1 immunoreactivity correlates with hormone secretion and histological parameters

**Figure 3:**
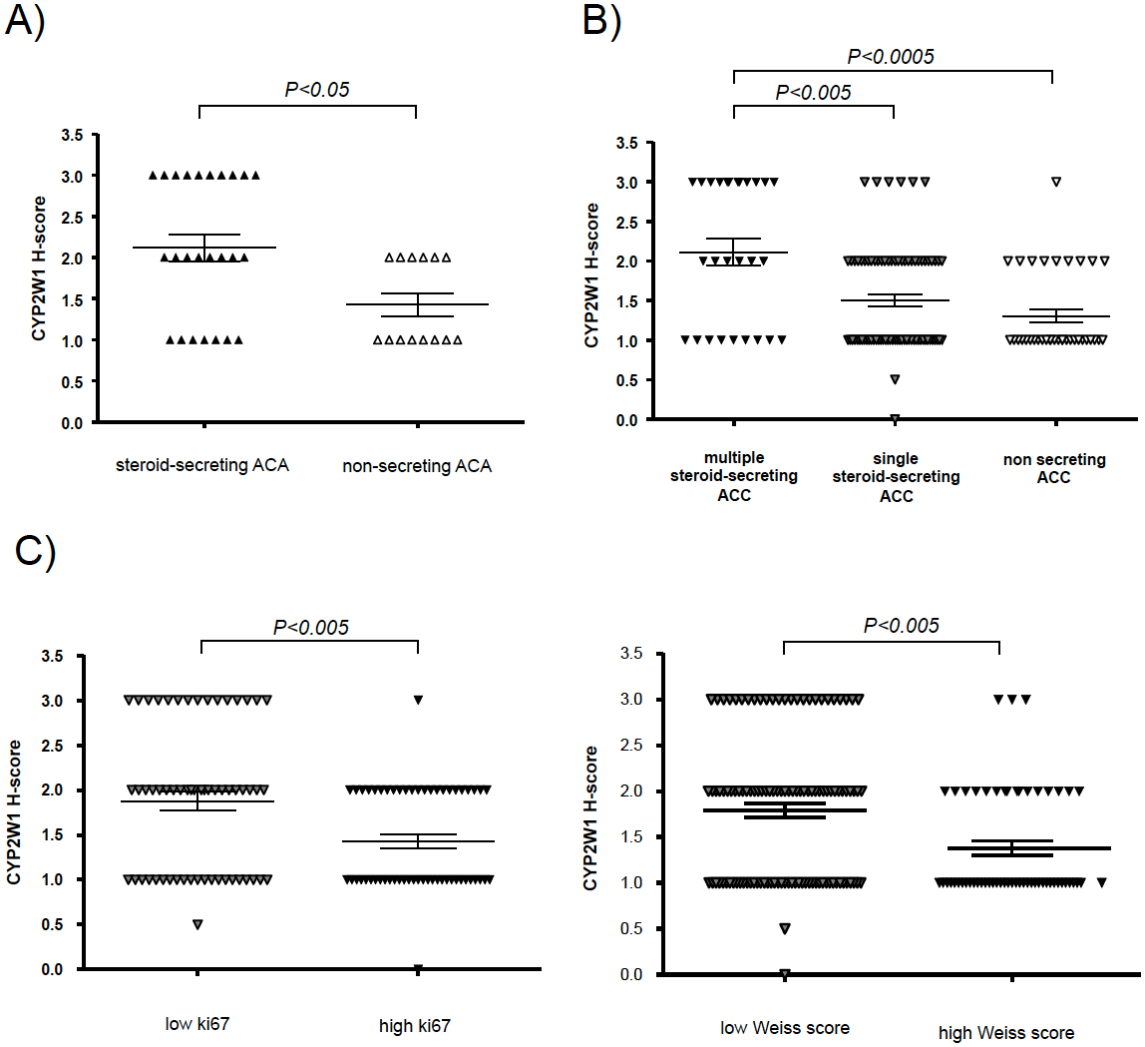
Relationship between CYP2W1 immunoreactivity and clinico-pathological parameters. A: Relationship with steroid (cortisol or aldosterone) secretion in adrenocortical adenomas with available hormonal data (ACA, n = 39). B: Relationship with steroid secretion in adrenocortical carcinomas with available hormonal data (ACC, n = 141). Multiple hormone secretion: androgen- and cortisol-secretion; single secretion: only androgen-, cortisol- or aldosterone-secretion. C: Relationship with the proliferation index (Ki67) in adrenocortical carcinomas (ACC, only samples deriving from primary surgery with known Ki67, n = 115). Legend: high Ki67>10% (median value), low Ki67≤10%. D: Relationship with Weiss score in adrenocortical carcinomas (ACC, only samples deriving from the primary surgery with known Weiss score, n = 171). Legend: high Weiss score>6 (median value), low Weiss score ≤6.

Steroid-secreting ACA (n = 25) exhibited higher CYP2W1 immunostaining than non secreting-tumors (n = 14, *P<0.05*, ***Figure 3A***), without any significant difference between cortisol- (n = 17) and aldosterone-secreting tumors (n = 8). Similarly, ACC samples with multiple hormone secretion had higher CYP2W1 immunoreactivity than those with single hormone secretion (only androgen, cortisol or aldosterone) or inactive ACC (***Figure 3B***). No significant difference was observed between androgen-, cortisol- or aldosterone-secreting tumors.

Considering only ACC samples obtained from primary surgery (n = 196), CYP2W1 immunoreactivity was significantly lower in tumors with a high Ki67 proliferation index (Ki67>10%, n = 59) than in those with low Ki67 (n = 56, *P<0.005*) and in those with a high Weiss score (Weiss score >6, n = 113) compared to those with low Weiss score (n = 58, *P<0.005,* ***Figure 3C and 3D*** and ***Table 1***).

**Table 1:** Relationship between CYP2W1 immunoreactivity and baseline clinical or pathological characteristics of patients with adrenocortical carcinoma (only tumour samples derived from primary surgery, n = 196).

	Low CYP2W1(H-score 0–1)	High CYP2W1(H-score 2–3)	P
N (%)	98 (50)	98 (50)	NS
Age – median (yrs)	47.0	49.4	NS
Sex – n male (%)	34 (47)	39 (53)	NS
Tumour size – median (cm)	11	13	NS
Tumour stage (ENSAT) – n (%)			
1–2	49 (57)	37 (43)	***<0.05***
3–4	37 (41)	53 (59)	
Weiss score - median	6	5	***<0.005***
Ki67 index – median (%)	16	11	***<0.005***
Overall survival – median (months)	43	53	0.45
DFS/TTP – median (months)	54	96	0.44

*P value with Chi-Square test among different response to treatments.

DFS = disease free survival, TTP = time to progression.

### CYP2W1 expression and clinical outcome

In a univariate analysis, CYP2W1 immunoreactivity was not associated with prognosis for both OS (n = 196 patients from first surgery, median 71.5 vs 43 months with high and low CYP levels, respectively, HR = 1.22, 95% CI = 0.8–1.8, *P = 0.33*) and DFS (n = 50 patients with complete tumor resection after first surgery, median 38 vs 27 months, HR = 1.42, 95% CI = 0.6–3.2, *P = 0.47*). When restricting the analysis to patients initially diagnosed with ENSAT stage 1 and 2, we observed only a slightly increased OS (n = 86, median 154 vs 110 months, HR = 1.59, 95% CI = 0.8–3.2, *P = 0.20*) and DFS for tumors with high CYP2W1 immunoreactivity (n = 27, median 64 vs 24 months, HR = 2.06, 95% CI = 0.7–6.2, *P = 0.20*).

### CYP2W1 immunoreactivity is associated with response to mitotane treatment

**Figure 4:**
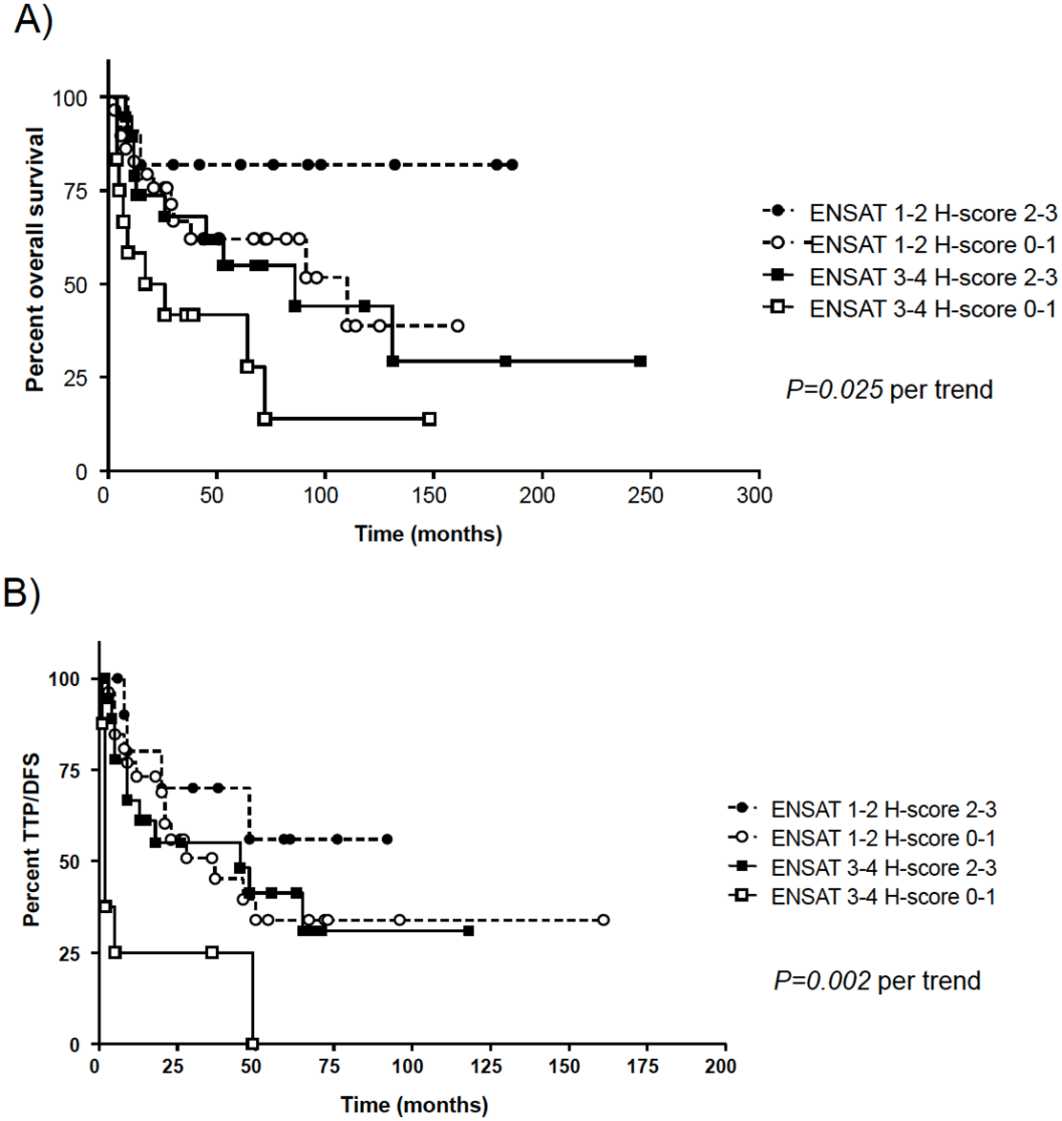
Impact of combined CYP2W1 immunohistochemistry and initial ENSAT tumor stage on clinical outcome in patients affected by adrenocortical carcinoma treated with mitotane only (n = 72, including both adjuvant and palliative intention). A: Overall survival; B: Time to progression (TTP).

When considering separately the 72 patients with ACC treated with mitotane only (either as adjuvant or palliative therapy), we observed a slightly longer OS (median 131 *vs* 72 months, HR = 1.61, *P = 0.17,* ***Table 2***) and time to progression (TTP, median 45 *vs* 24 months, HR = 1.37, *P = 0.29* ***Table 2***) for tumors with high CYP2W1 immunoreactivity. After adjusting the log-rank test analysis for the ENSAT tumor stage at diagnosis, the associations became statistically significant (OS: *P = 0.025*, and TTP: *P = 0.002,* ***Figure 4A and 4B***). At multivariate analysis including CYP2W1 levels, ENSAT tumor stage, and Ki67 proliferation index, the impact of CYP2W1 expression on both OS and TTP was statistically significant (HR = 3.9, *P = 0.009* and HR = 4.9, *P = 0.001*, respectively, ***Table 2***). In contrast, no correlation between CYP2W1 and the clinical outcome was observed in ACC patients who underwent only follow up (no pharmacological treatment, n = 39) or who received different systemic cytotoxic chemotherapy (n = 64).

**Table 2:** Relationship between CYP2W1 immunoreactivity and clinical outcome of patients with adrenocortical carcinoma treated with mitotane only (n = 72).

		Univariate analysis				Multivariate analysis	
	n	Median survival(months)	HR (95% CI)		P	HR (95% CI)	P
**Overall survival**							
Tumour stage (ENSAT)			2.01		*0.040*	3.5	***0.009***
1–2	40	NR	(1.0–4.2)			(1.4–9.2)	
3–4	31	53					
Ki67 index (%)*			1.84		*0.16*	1.52	*0.33*
<7	25	NR	(0.8–4.3)			(0.6–3.6)	
>7	32	110					
CYP2W1 immunoreactivity			1.61		*0.17*	3.9	***0.009***
Low	41	72	(0.8–3.2)			(1.4–11.0)	
High	30	131					
**TTP/DFS**							
Tumour stage (ENSAT)			1.83		*0.08*	3.3	***0.012***
1–2	40	48	(0.9–3.6)			(1.3–4.2)	
3–4	31	21					
Ki67 index (%)*			3.03		*0.007*	2.0	*0.088*
<7	25	NR	(1.3–6.8)				
>7	32	21					
CYP2W1 immunoreactivity			1.53		*0.20*	4.88	***0.001***
Low	41	23	(0.8–2.3)				
High	30	48					

HR = hazard ratio, 95% CI = 95% confidence interval, NR = not reached, DFS = disease free survival, TTP = time to progression.

*cut off value for Ki67 index: median value in the evaluated population.

**Figure 5:**
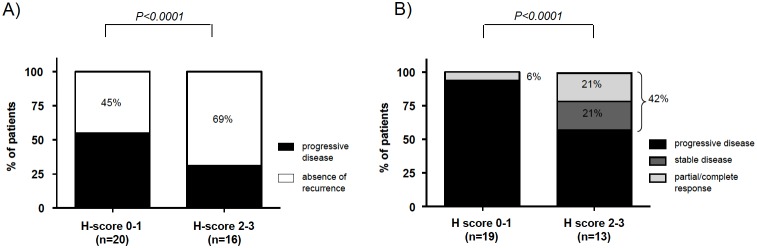
Relationship between CYP2W1 immunoreactivity and response to therapy with mitotane only, according to treatment intention. A: Response to therapy in patients treated in an adjuvant setting (n = 36). B: Objective response to therapy in patients treated in a palliative setting (n = 32).

We next examined mitotane treated patients according to treatment intention. A trend to better OS was observed in case of high CYP2W1 immunoreactivity in both the 36 patients treated adjuvantly (HR = 3.6, 95% CI = 0.7–18.1, *P = 0.11,* ***Figure 5A***) and in the 36 patients treated in a palliative setting (median survival 45 vs 19 months, HR = 1.4, 95% CI = 0.65–3.2, *P = 0.37,* ***Figure 5B***). Moreover, we investigated the objective response to treatment in both subgroups. Of note, it was significantly better in patients with high CYP2W1 expression (absence of disease recurrence in 69% *vs* 45% of patients treated adjuvantly, *P = 0.001,* complete/partial response or stable disease in 42% *vs* 6% of patients treated with palliative intention, *P = 0.0019,* ***Figure 5C and 5D***).

No impact of CYP2W1 immunoreactivity on response to treatment was observed in the subgroup of ACC patients treated with different systemic cytotoxic chemotherapies (n = 64).

## Discussion

This large study on CYP2W1 expression in adrenal glands reveals several key findings: we observed a high expression of CYP2W1 in the majority of adrenocortical tumors, comprising both adenomas and carcinomas, but also in normal adult adrenal tissue. Furthermore, CYP2W1 immunoreactivity in adrenocortical tumors was associated with hormonal activity, with a more differentiated phenotype and in ACC with better response to mitotane therapy.

CYP2W1 has been classified as an orphan human cytochrom P450 enzyme, as its physiological substrate is still unknown. Of major interest is that high CYP2W1 expression is supposed to be restricted to foetal tissues and to some cancers [11], [12], [13], [15]. However, expression of CYP2W1 in normal adult tissues is still under debate. Some papers reported that CYP2W1 is not expressed in normal adult tissues using dot blot experiments in a panel of normal tissues including one adrenal probe [6], [15], but to date no systematic investigation has been performed. In contrast, other studies found an mRNA expression of CYP2W1 also in several normal adult tissues including spleen, testis, lung, pancreas, placenta, ovary, thymus, prostate, colon, and small intestine [16]. In agreement with these observations, the human Protein Atlas web-site describes also CYP2W1 protein expression in normal tissues, such as Leydig cells, pancreas, stomach, myocytes, neuronal cells, thyroid and adrenal (www.proteinatlas.org; Version 11.0, [26]. In our investigation, very low mRNA expression and low CYP2W1 immunoreactivity was detected in non-adrenal normal samples, while interestingly high levels were definitively found in normal adult adrenal glands.

The expression of CYP2W1 in normal adrenal raises the question of a specific function in adrenal physiology. In our adrenocortical tumors, CYP2W1 was expressed at higher levels in hormonally active tumors (steroid secreting) than in inactive tumors, suggesting a possible relationship between the CYP2W1 expression and the hormone production, which needs to be further evaluated.

Expression of CYP2W1 mRNA has been reported previously in a small number of adrenal tumors, but no details of the respective tumor entities were reported [6], [15]. While in colon cancer expression of CYP2W1 occurs in about 30% of cases and is known to be associated with a poor prognosis, in adrenocortical tumors we observed a high expression in both benign and malignant tumors. Furthermore, in ACC it was associated with a lower Ki67 index and a lower Weiss score, suggesting rather a more differentiated phenotype. Hence, in striking contrast to the findings in colon cancer, CYP2W1 expression in ACC was not associated with poor prognosis. Instead, higher CYP2W1 levels were associated with a slightly better outcome in patients with early ENSAT stages, suggesting that CYP2W1 may be a possible favourable prognostic marker.

We are aware that demonstrating expression of CYP enzymes by immunohystochemistry invariably raises questions concerning the specificity of the antibodies used. However, the antibody that we used for immunostaining (**Ab #1**) has been raised against a peptide sequence highly specific for CYP2W1 among the CYP family. Furthermore, we found good agreement between the degree of mRNA and protein expression (***Figure S3***) and, finally, at Western Blot analysis with three different antibodies raised against different regions of CYP2W1 we consistently detected a specific band corresponding to CYP2W1 protein in positive controls from transfected cells.

As it has been demonstrated that CYP2W1 is able to activate a variety of chemical carcinogens to genotoxic compounds, CYP2W1 has become a promising tool in targeted therapy for malignancies expressing this enzyme, as it may allow to specifically targeting tumor cells with limited systemic toxicity. Accordingly, some anticancer compounds have been developed, which are selectively bioactivated by CYP2W1 into potent cytotoxic drugs, such as AQ4N [27], 5F-203 and GW-610 [12], and duocarmycin bioprecursors [28], [29]. This approach has recently been studied both *in* *vitro* and *in* *vivo* demonstrating that the duocarmycin analog ICT2706 is converted by tumor cells into a potent cytotoxin inhibiting the growth of human colon cancer xenografts [28], [29]. Of particular interest is the observation of a bystander effect affecting adjacent cells not expressing CYP2W1 by the toxins generated in CYP2W1 expressing cells, suggesting a relevant therapeutic potential of an anti-tumor strategy exploiting bioactivation by CYP2W1. Current treatment options for ACC are still largely disappointing [2], [30], [31] and the high expression of CYP2W1 in many ACCs indicates that CYP2W1 might become an intriguing target for tumor specific treatment. We then asked whether CYP2W1 expression might be related to the response to different pharmacological treatments for ACC. We did not observe any relationship between the CYP2W1 expression and the response to cytotoxic drugs, but surprisingly we found a significant impact of CYP2W1 levels on the efficacy of the therapy with mitotane, which is the only drug currently approved for the treatment of advanced ACC. This finding is particularly intriguing because specific biomarkers predicting the response to mitotane are still lacking, except for the gene expression of ribonucleotide reductase large subunit 1 (RRM1, [32]). Furthermore, it has been suggested that mitotane needs to be activated in adrenocortical tissue to exert its adrenotoxic effects [33], [34], but the precise molecular mechanisms by which mitotane exerts its adrenolytic effects are still largely unknown. Intriguingly, in patients treated with mitotane alone, we observed a significantly better overall survival in patients with ENSAT tumor stage 1 or 2 (median survival: 110 vs 70 months, HR = 3.22). An objective response to mitotane treatment was more likely in patients with high CYP2W1 than in those with low CYP2W1 immunoreactivity, both in a palliative and an adjuvant setting. On the other hand, no impact of CYP2W1 on clinical outcome has been observed in tumors from patients who underwent only follow up (no medication) or were treated with systemic cytotoxic chemotherapy. These findings indicate that CYP2W1 expression may serve as new molecular marker for predicting the response to mitotane therapy, both as adjuvant or palliative option, in patients with ACC. Moreover, they could imply a potential role of the enzyme CYP2W1 in the intra-adrenal metabolism of mitotane. Oxidation by CYP2W1 might contribute to the activation of mitotane and at least in part explain its specific activity in malignancies of the adrenal cortex. Obviously, functional experiments are needed to establish a role of CYP2W1 for the cytotoxic effects of mitotane in adrenal cells.

The high expression of CYP2W1 in normal adrenal glands warrants further attention since it may lead to undesirable drug effects when prodrugs are used that are activated by CYP2W1. Adrenocortical drug activation may affect normal adrenal function and carry a risk of adrenal insufficiency. Although such an effect has not been reported in mice with human colon cancer xenografts receiving duocarmycin analogs, specific investigations concerning steroid secretion or adrenal morphology have not been performed. On the other hand, mitotane therapy for ACC is also associated with adrenal insufficiency and hydrocortisone replacement is used to readily compensate the adrenotoxic effects of mitotane on steroid secretion.

In summary, a high mRNA expression of CYP2W1 was observed in both normal and neoplastic adrenal glands and CYP2W1 immunoreactivity was associated with increased hormone-production and with a more differentiated phenotype. Furthermore, CYP2W1 levels were related to a better response to treatment with mitotane, rendering it a possible prognostic marker for patients who undergo mitotane therapy. The expression of CYP2W1 in ACC makes this enzyme a promising drug target, by taking advantage of its ability to convert nontoxic prodrugs (i.e. duocarmicyn precursors) into highly potent tumor toxins.

## Supporting Information

Figure S1 **Results of Western Blot analysis.** Western Blot analysis in three human cortisol-secreting adrenocortical adenomas, in a negative control (NC) and in two positive controls (PC1 and PC2, derived from HEK cell lines transfected with CYP2W1, [15] with different antibodies (see Material and Methods): A: CYP2W1 Ab from Thermo Scientific (**Ab #1**, N-terminal, 1∶50, 5 min), B: CYP2W1 Ab provided as a gift by Karolinska Institute (**Ab #2**, C-terminal, 1∶1000, 20 min), C: CYP2W1 Ab from Santa Cruz (internal region, 1∶200, 4 min).
(TIF)

Figure S2 **Relationship between CYP2W1 mRNA and immunoreactivity in 25 adrenocortical tumors** (8 adenomas and 17 carcinomas). The relative mRNA levels are expressed as quartiles of the ΔCT values, while the CYP2W1 immunoreactivity is expressed as H-score (see materials and methods section).
(TIF)
