# Supplementary figures and images for: CYP2W1 Is Highly Expressed in Adrenal Glands and Is Positively Associated with the Response to Mitotane in Adrenocortical Carcinoma
Source: PLoS One. 2014 Aug 21;9(8):e105855. doi: 10.1371/journal.pone.0105855 (PMC4140842; doi:10.1371/journal.pone.0105855)

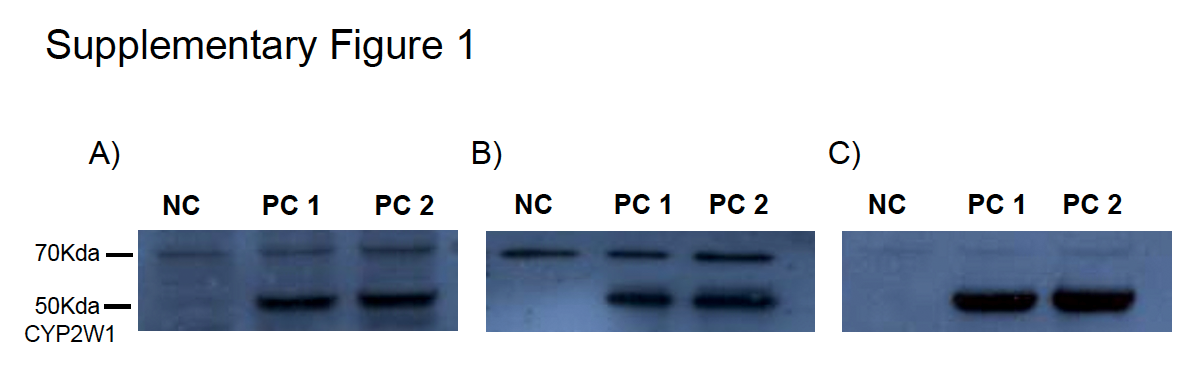

Supplement: Figure S1 — Results of Western Blot analysis. Western Blot analysis in three human cortisol-secreting adrenocortical adenomas, in a negative control (NC) and in two positive controls (PC1 and PC2, derived from HEK cell lines transfected with CYP2W1, [15] with different antibodies (see Material and Methods): A: CYP2W1 Ab from Thermo Scientific (Ab #1, N-terminal, 1∶50, 5 min), B: CYP2W1 Ab provided as a gift by Karolinska Institute (Ab #2, C-terminal, 1∶1000, 20 min), C: CYP2W1 Ab from Santa Cruz (internal region, 1∶200, 4 min). (TIF) [file pone.0105855.s001.tif]

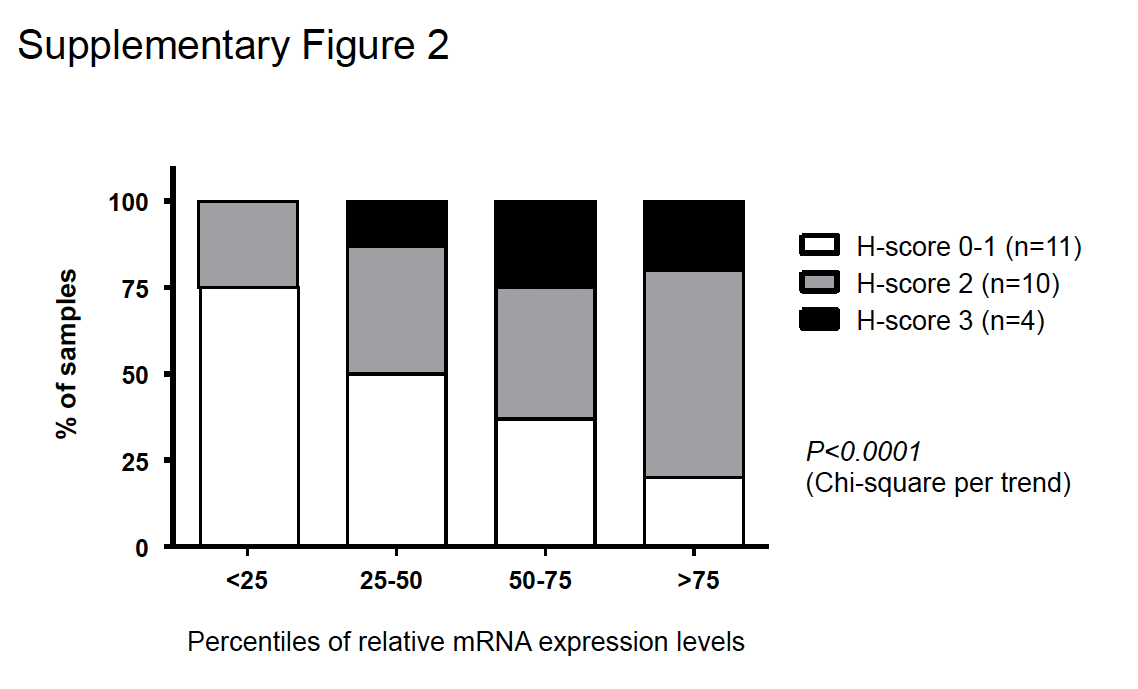

Supplement: Figure S2 — Relationship between CYP2W1 mRNA and immunoreactivity in 25 adrenocortical tumors (8 adenomas and 17 carcinomas). The relative mRNA levels are expressed as quartiles of the ΔCT values, while the CYP2W1 immunoreactivity is expressed as H-score (see materials and methods section). (TIF) [file pone.0105855.s002.tif]
